# Pancreaticopleural fistula in a child with chronic pancreatitis harboring compound SPINK1 variants

**DOI:** 10.1186/s12876-021-01842-1

**Published:** 2021-06-12

**Authors:** Teera Kijmassuwan, Prapun Aanpreung, Varayu Prachayakul, Prakarn Tovichien

**Affiliations:** 1 Division of Gastroenterology, Department of Pediatrics, Siriraj Hospital, Mahidol University, Bangkok, Thailand; 2 Division of Pulmonology, Department of Pediatrics, Siriraj Hospital, Mahidol University, Bangkok, Thailand; 3 Division of Gastroenterology, Department of Internal Medicine, Siriraj Hospital, Mahidol University, Bangkok, Thailand

**Keywords:** Pancreaticopleural fistula, Chronic pancreatitis, *SPINK1* mutation, Endoscopic management, Case report

## Abstract

**Background:**

Pancreaticopleural fistula (PPF) is a rare complication of chronic pancreatitis (CP) that requires a high index of clinical suspicion in the patient who presents with a pleural effusion. Visualizing the fistula tract from the pancreatic duct to the pleural space by radiological imaging provides confirmation of this complication.

**Case presentation:**

A 9-year-old boy who presented with massive right pleural effusion secondary to PPF, a complication of CP from a genetic mutation involving two mutations of *SPINK1*. We successfully managed the case with by endoscopic pancreatic duct stent placement after failure of conservative treatment approaches.

**Conclusions:**

PPF is a rare but serious complication of CP in all ages. The diagnosis of PPF in children requires a high index of clinical suspicion and should be considered in the differential diagnosis of massive pleural effusion where pancreatic pathology is present. A high level of pleural fluid amylase and the results from radiological imaging when the patients have symptoms play essential roles in the diagnosis of PPF. Currently, Magnetic resonance cholangiopancreatigraphy (MRCP) is the imaging modality of choice. Endoscopic therapy and surgery are treatment options for patients who do not respond to conservative therapy.

## Background

Chronic pancreatitis (CP) is the irreversible change of the pancreatic parenchyma, which can be caused by many contributing risk factors. More than 67% of pediatric CP is caused by genetic mutations [1]. Pleuropulmonary manifestations, such as pleural effusion, and pancreaticopleural fistula (PPF), are associated with significant morbidity and mortality. In adults, PPF is usually caused by alcohol-induced CP and acute necrotizing pancreatitis, which has an incidence of 0.4% in patients presenting with CP [2, 3]. In children, there is insufficient data on the incidence and causes of PPF, such as acute pancreatitis (blunt trauma and unknown causes) and CP (ductal anomalies, *PRSS1* mutation, and unknown causes) [4–11]. PPF may initially present with a shortness of breath, dyspnea, or chest tightness, with or without a history of abdominal pain or steatorrhea. Elevated pleural fluid amylase suggests the presence of PPF. Computer tomography (CT) of the abdomen or magnetic resonance cholangiopancreatography (MRCP) is the gold standard for diagnosis. Conservative, surgical, or endoscopic management treatment modalities are available [12–15]. However, endoscopic management of pediatric patients with PPF remains uncommon [8, 9, 11, 15]. This report presented a young child whom failed conservative and needed to further management of PPF.

## Case presentation

**Fig. 1:**
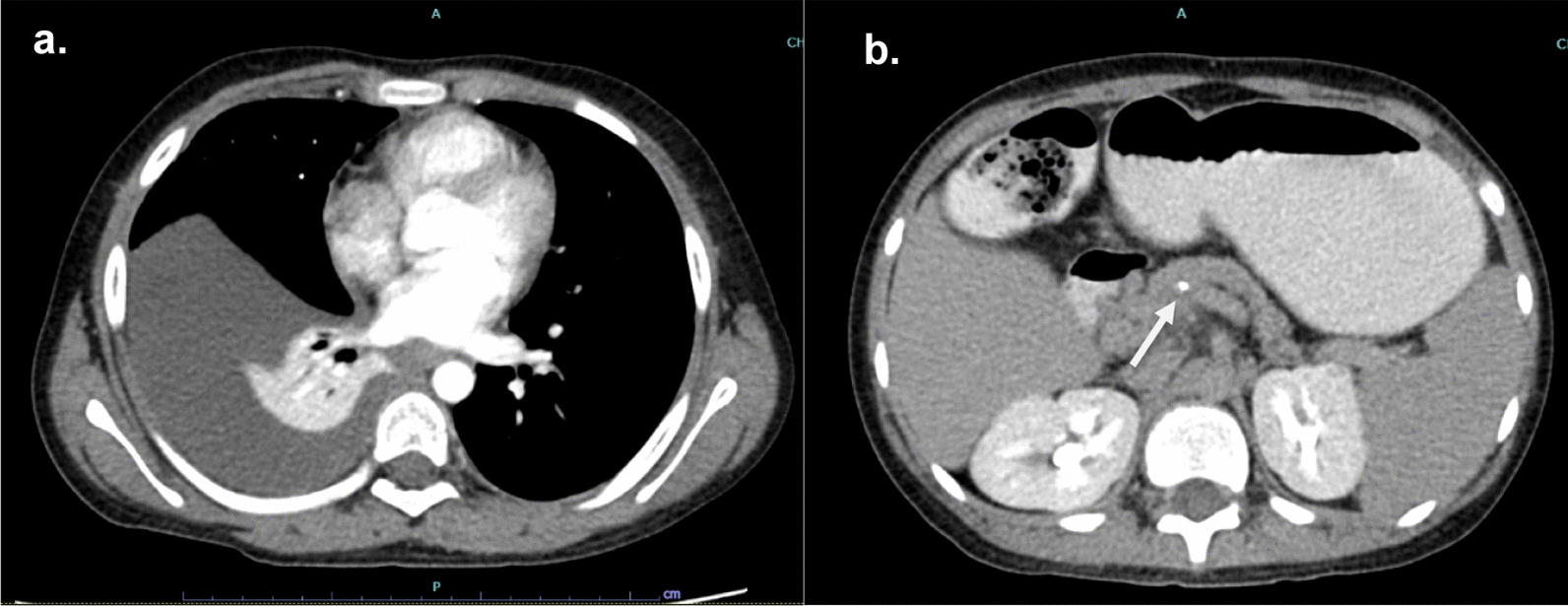
Initial CT scan reveals **a** pleural effusion and **b** calcification in the head of pancreas with dilatation of pancreatic duct (arrow)

A 9-year-old boy was referred to Siriraj Hospital with recurrent abdominal pain and a three-week history of breathing difficulty. His first episode of abdominal pain appeared eight months prior and the symptoms were worsening. The patient had a normal perinatal history, developmentally age appropriate, and a negative family history of chronic pancreatitis. His height was 139 cm (75th percentile) and his weight was 28 kg (50th percentile). He was taken to the primary care hospital because he had fever and dyspnea for two days. Physical examination found a decrease in breath sounds at the right lung with dullness on percussion and mild tenderness at the epigastrium. Chest radiography showed massive right pleural effusion. The pleural fluid analysis revealed an exudative pattern with leukocytes count of 5,900 cell/L (70% neutrophil) and protein of 5.1 g/dL (serum protein 7.2 g/dL). A CT of the chest was sent to confirm massive right pleural effusion and to define the associated pathology but calcification of the pancreatic parenchyma suggestive of CP was accidentally found (Fig. 1). Further investigation of CP, serum amylase and lipase were 411 U/L and 532 U/L, respectively. Calcium, triglycerides, and blood sugar were normal. The patient was referred to our hospital for evaluation and treatment.

**Fig. 2:**
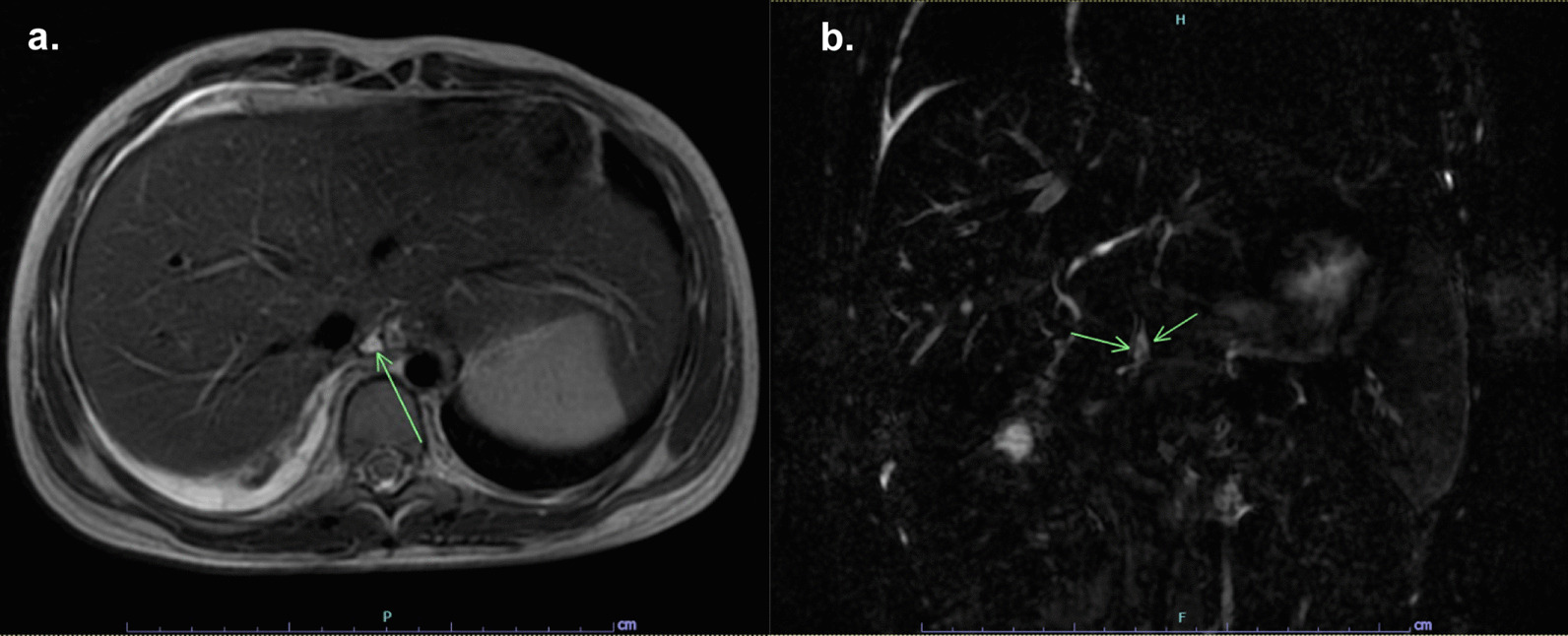
MRCP demonstrates dilatation of the pancreatic duct and a high-signal intensity structure arising from the proximal pancreatic duct reaching up to the right pleural cavity. **a** Transverse and **b** coronal view

**Fig. 3:**
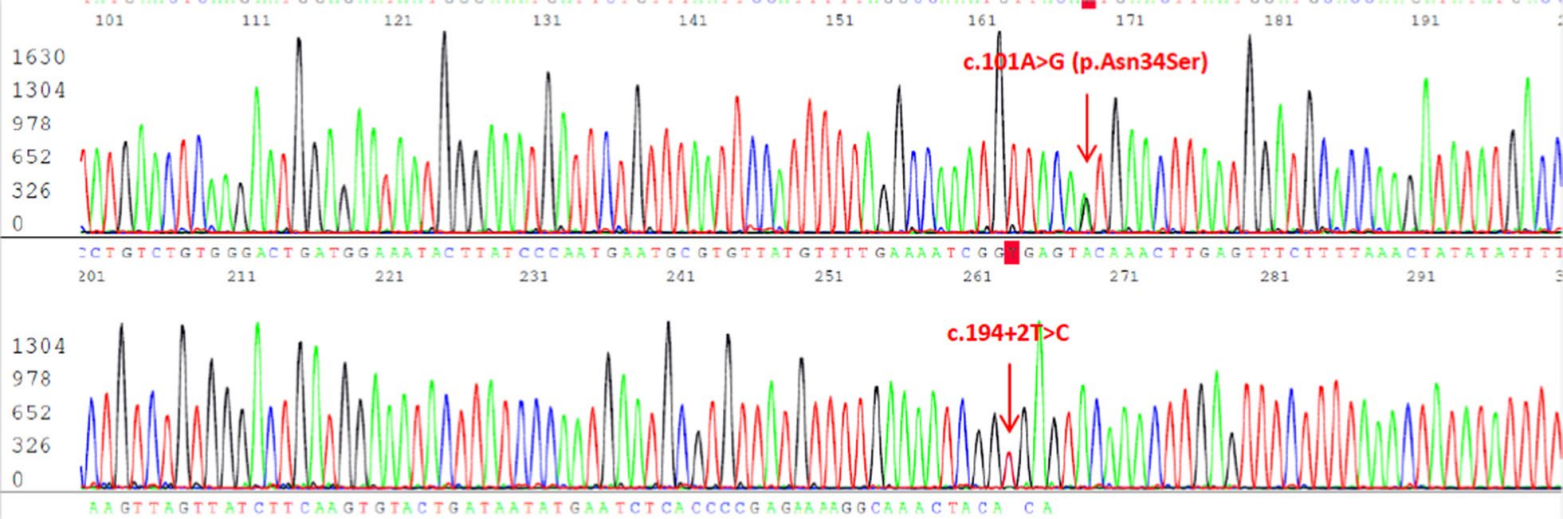
Two heterozygous variants in SPINK1, c.101A>G (p.Asn34Ser) and c.194+2T>C in this patient

MRCP revealed atrophy of the body and tail of the pancreas with multifocal calcifications and chain of lakes dilations of the distal pancreatic duct, compatible with CP. There was a high-signal intensity structure arising from a proximal pancreatic duct reaching up to the right pleural cavity, which raised the suspicion of PPF (Fig. 2). At this point the pleural effusion had decreased, so we could not perform pleural fluid analysis. The patient was started on conservative management with intravenous octreotide at up to 1 mcg/kg/hr, intravenous hydration, parenteral nutrition, and kept NPO. PCR amplification of the *PRSS1* and *SPINK1* genes was performed by direct DNA sequencing to find the cause of CP. The results were negative for *PRSS1* mutation, but two heterozygous *SPINK1* variants were identified: a pathogenic splicing mutation (c.194+2T>C) and a common missense variant (c.101A>G, p.Asn34Ser) (Fig. 3). The patient’s abdominal pain and dyspnea well responded to the treatment and the chest radiography showed a decrease in the right pleural effusion, so octreotide was tapered and then discontinued after three days. The patient was admitted in total for three weeks and then discharged to home with antioxidants (selenium, and vitamins A, C, and E).

**Fig. 4:**
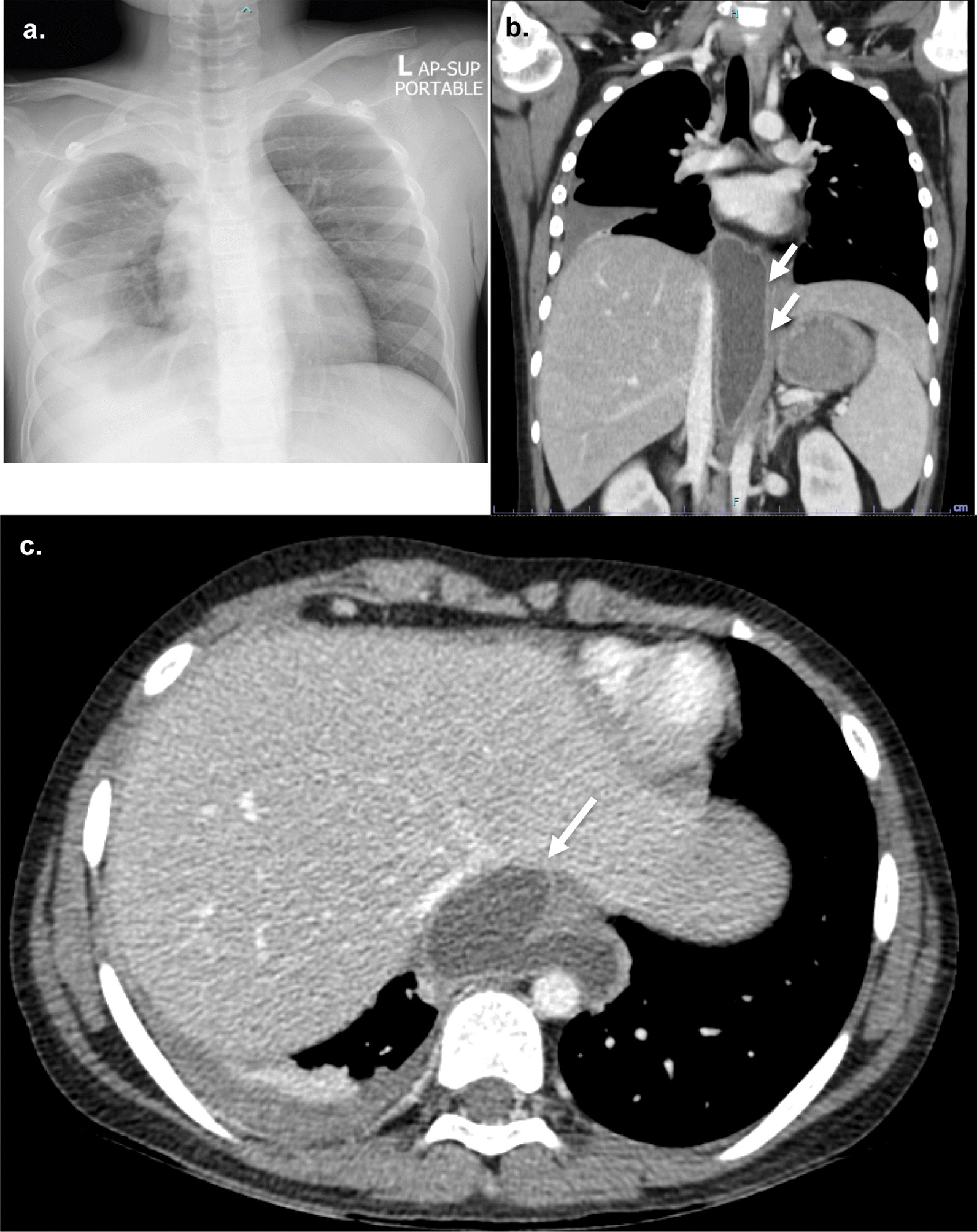
Chest radiography and CT scan from the second admission **a** massive pleural effusion, **b** transverse and **c** coronal plane of CT abdomen demonstrate a mediatinal pseudocyst and PPF arise from proximal pancreatic duct (arrow) reaching up to right pleural cavity

**Fig. 5:**
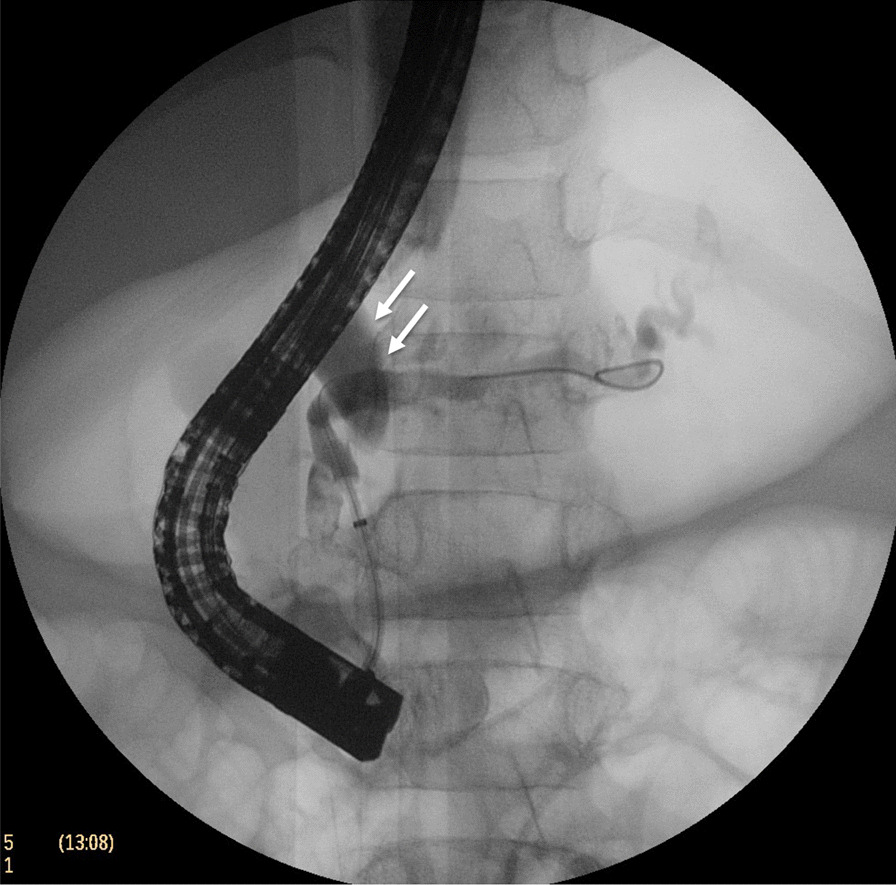
ERP demonstrated irregular and dilated of pancreatic duct. Short segment stricture and disruption of pancreatic duct (arrow) connected to pleural space was showed at pancreatic neck level

One week after discharge, the patient developed fever, cough, and mild dyspnea. Physical examination at the primary care hospital found decreased breath sound in the right lung. Chest radiography demonstrated a large right pleural effusion (Fig. 4A). Thoracentesis was performed, returning 170 ml of red effusate. Pleural fluid analysis revealed an amylase level 42,798 U/L, and the bacterial culture was negative. Serum amylase and lipase were 507 and 521 U/L, respectively. The patient was again referred to our hospital. Respiratory tract infection was worked up and influenza A infection was detected by nasal swab PCR. The patient’s abdominal pain returned and the laboratory found an increase in serum amylase and lipase 632 and 713 U/L, respectively. CT of the chest and abdomen demonstrated a pleural effusion with enlarged PPF (Fig. 4B) compared to the previous MRCP. Bowel rest, continuous intravenous octreotide infusion, and total parenteral nutrition were again used to treat the pleural effusion, but this time the patient had persistent gastrointestinal and respiratory symptoms. Endoscopic retrograde pancreatography (ERP) confirmed a duct disruption at the pancreatic neck with a fistula connected to the posterior mediastinum but cannot demonstrate the pseudocyst. Without dilatation of the pancreatic duct, a 7Fr plastic stent was placed in the main pancreatic duct across the leakage site (Fig. 5). After placement of the stent, the patient’s clinical and chest radiography results improved and he was discharged one week later. The stent was removed after six months. At 6 months post-stent removed, the patient was had no chest or abdominal symptoms and the chest X-ray (CXR) was negative for pleural effusion.

## Discussion and conclusions

Genetic mutation is the most common cause of pediatric CP [1]. The four common gene mutations are cationic trypsinogen gene (*PRSS1*), cystic fibrosis transmembrane conductance regulator gene (*CFTR*), serine protease inhibitor Kazal type 1 gene (*SPINK1*), and chymotrypsin C gene (*CTRC*) [16]. Mutations in the *SPINK1* gene are associated with a change in the defense mechanism against trypsin activation and have been reported to lower the threshold for pancreatitis in the presence of other genetic or environmental factors [17-19]. The frequency of *SPINK1* mutations in idiopathic CP varies from 6.4 to 40% [20, 21]. One study reported the median age at onset of symptoms to be 20.1 years old and did not involve complications of PPF [20]. However, an early age of onset and severe disease may be caused by compound *SPINK1* mutations [22]. Currently, there is no definitive management for the *SPINK1* mutation; close monitoring for complications such as chronic pain, exocrine pancreatic insufficiency, and diabetes mellitus is required. Antioxidants have been used to decrease free radical injury, oxidative stress, and for pain relief. However, little evidence exists to support the hypothesis that antioxidants can prevent disease progression or reduce pain in children [23].

Pancreaticopleural fistula is a rare complication of chronic pancreatitis. A leakage of pancreatic fluid from disruption of the pancreatic pseudocyst to the pleural cavity is the cause of PPF. PPF and pleural effusion from other causes usually have similar clinical manifestations, such as a shortness of breath, dyspnea, or orthopnea. The diagnosis can be confirmed by pleural effusion analysis and by the presence of a pseudocyst and/or fistula in the radiographic imaging. In the present case, the high levels of amylase in pleural fluid than in the serum level suggested this diagnosis [13-15].

A fistula between the pancreatic duct and pleural space confirms of diagnosis [3]. Currently, MRCP is the technique of choice because of it is less invasive and more sensitive than CT and ERCP [2]. However, the time of imaging and clinical data of the patient should be correlated.

A conservative treatment approach initially involves pleural fluid drainage, octreotide, fasting, and parenteral nutrition [12-14]. Overall, 30–65% of patients with PPF respond well to conservative treatment [2]. There is no consensus on the duration of conservative treatment. If the patient does not respond well after two or three weeks of treatment, or if symptoms progress, interventional therapies such as ERP and surgical management should be considered. Endoscopic retrograde pancreatography with stent placement is preferred in adults because it is less invasive, but only a few successful cases in children have been reported [7, 11, 24]. Decreasing the ductal pressure and sealing the leakage site are the main benefits of the ductal stent. Post-ERCP complications such as infection, acute pancreatitis, bleeding, or perforation occur in 7–25% of cases [14]. Surgical management by pancreatic resection or formal operative pancreatic ductal drainage procedures is indicated for patients with ductal disruption located near the tail of the pancreas, complete obstruction of the pancreatic duct, or failed ERCP treatment.

PPF is a rare but serious complication of CP. The most common risk factor of CP in children is genetic mutations. The diagnosis of PPF should be considered in the differential diagnosis of massive pleural effusion where pancreatic pathology is present. The diagnosis can be confirmed by a high level of amylase in pleural fluid and by the presence of a pseudocyst and/or fistula in the radiographic imaging. Endoscopic therapy in children who do not respond to conservative therapy is safe and effective.

## Data Availability

This data are available from the authors upon reasonable request and with the permission of the institution.

## Abbreviations

CP: Chronic pancreatitis
CT: Computer tomography
ERCP: Endoscopic retrograde cholangiopancreatography
MRCP: Magnetic resonance cholangiopancreatography
PPF: Pancreaticopleural fistula
SPINK1: Serine protease inhibitor Kazal type 1
